# Social Distancing Requirements and the Determinants of the COVID-19 Recession and Recovery in Europe

**DOI:** 10.1007/s10272-020-0935-8

**Published:** 2020-12-01

**Authors:** Christian Dreger, Daniel Gros

**Affiliations:** 1 Wirtschaftswissenschaftliche Fakultät (Wiwi), European University Viadrina, Große Scharrnstraße 59, 15230 Frankfurt (Oder), Germany; 2 Centre for European Policy Studies (CEPS), 1 Place du Congrès, 1000 Brussels, Belgium

## Abstract

Social distancing restrictions, as measured by the Oxford stringency indicator, constitute the one variable that is most tightly correlated with the recession and recovery across EU member states.

The COVID-19 pandemic led to an unprecedented decline in economic activity. Starting in February 2020, policymakers around the globe introduced emergency measures to slow down the spread of the virus, such as social distancing and the cancellation of events, limitations to mobility and travel, and the shutdown of large parts of the economy, including firms, workplaces and schools.

Many restrictions introduced in March were gradually lifted as the number of new infections decreased. Starting in early summer, however, infections were on the rise again, partially driven by gatherings at local virus hotspots and the summer holidays. The rise is particularly striking in Spain, where the number of new infections exceed the

* This research has received funding from the European Union's Horizon 2020 research and innovation program "PERISCOPE: Pan European Response to the ImpactS of COvid-19 and future Pandemics and Epidemics", under the grant agreement No. 101016233, H2020-SC1-PHE_CORONAVIRUS-2020-2-RTD.

Figure 1
**Number of new COVID-19 infections and deaths in large EU countries, 2020**

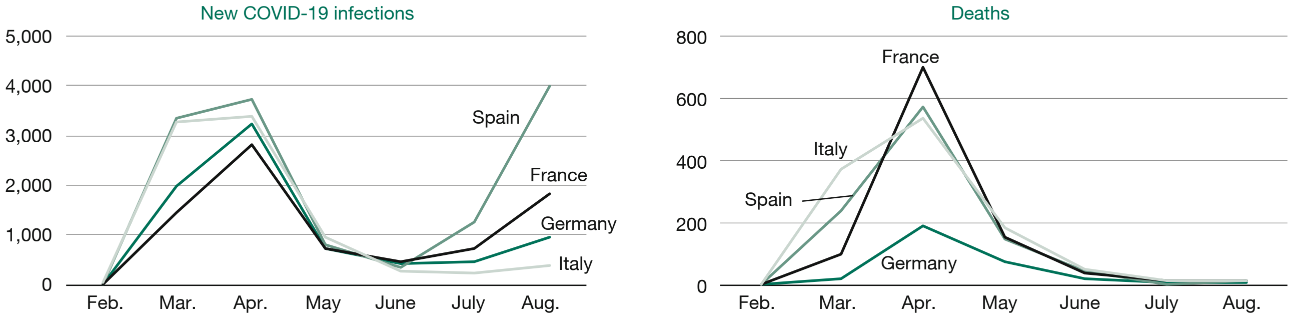

Source: John Hopkins Coronavirus Resource Center, Johns Hopkins University, Baltimore.

previous peak from April. Despite the increase of new infections, the number of deaths has remained relatively low (Figure 1 shows data for the four largest EU countries).

A key issue at this point is what effect the resurgence of infections will have on the economy. The need to 'flatten the curve' justified the harsh restrictions ('lockdown') imposed in March/April of 2020 (Baldwin and Weder di Mauro (2020a, b). The situation is different in the autumn of 2020, with far fewer fatalities as shown in Figure 1. The question at this point is how to disentangle the impact of different factors on the economy, such as infections, deaths, mobility and social distancing restrictions.

The appropriate design of policies is critical, as massive losses can be involved. However, empirical evidence on the impact of the respective measures is scarce. Several studies discuss the impact of non-pharmaceutical interventions on the state of the pandemic, such as the growth of infections in OECD member states (Pozo et al., 2020) or the mean decline in reproduction rates in a cross section of 41 countries (Brauner et al., 2020). A general result is that many different types of interventions are found to be successful in reducing the spread of the virus (Flaxman et al., 2020). However, there are important differences: While restrictions on gatherings, mask-wearing, school and workplace closures and testing ratios (tests per 1,000 citizens) appear to be effective to control for the transmission of the coronavirus, stay-at-home policies are less relevant.

The key challenge for policymakers is managing the trade-off between the severity of the lockdown measures and their effects on the diffusion of the virus (Eichenbaum et al., 2020). A few studies have examined the impact of restrictions on the economy, although from different angles. Bodenstein et al. (2020) stressed that the absence of social distancing policies may amplify the economic costs over longer time intervals. To lower the costs in economic terms, social distancing should be skewed to non-essential industries and professions that can be performed from home. Due to input-output linkages, however, even non-targeted industries could be affected. Getachev (2020) has argued that voluntary social distancing is important for both flattening the infection curve and minimising damage to the economy. According to Laeven (2020), producers of intermediates tend to be more affected by the crisis if they sell their output to industrial sectors restricted by social distancing. Following Barro et al. (2020), the losses in output and consumption attributed to the coronavirus are more pronounced than in the Spanish flu, even if further outbreaks of the virus are avoided.

A problem with all of these studies is that none of them utilise actual economic data for the pandemic period, as the information is available only with a delay. In contrast to previous studies, this paper explores the relation between non-pharmaceutical interventions and the economy using actual data. We propose this first analysis using actual economic data as a starting point for further research, which could take into account other factors, e.g. policy spillovers and the impact of the global cycle, in a more systematic way. The correlations we uncover are, however, suggestive of a pattern that should be of interest to policymakers.

The proceeding section of this paper presents two measures of social distancing, the Oxford stringency index and the Google mobility indicator. This is followed by a discussion of economic indicators. Besides industrial production, the economic sentiment indicator proposed by the European Commission serves as a proxy for contemporaneous GDP.

Figure 2
**Indicators of social distancing and the lockdown**

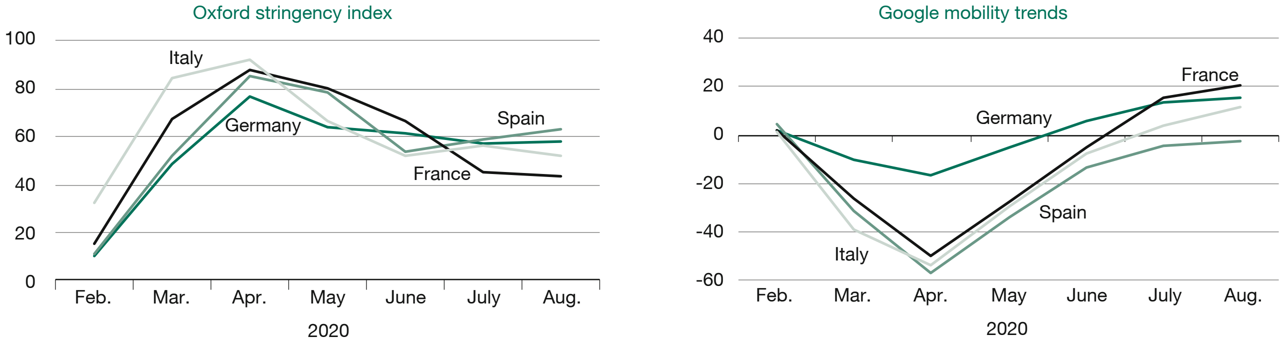

Note: The Oxford stringency index summarises information from different lockdown components. The Google mobility indicator is based on aggregated movements for different places.

Sources: Oxford COVID-19 Government Response Tracker, 2020; Google COVID-19 Community Mobility Reports, 2020.

Many previous studies have looked at cross sections of countries and did not control for unobserved heterogeneity. In contrast, our estimation is based on panel models with country fixed effects.[1] Specific policy measures are also distinguished. A main finding is that stay-at-home regulations show the strongest relation with the fall in GDP and industrial production. As medical studies have concluded that stay-at-home restrictions are less important for the spread of the virus, potential future interventions should focus primarily on other components of social distancing, such as restrictions on public events and public gathering.

## Measuring social distancing

Social distancing refers to the many changes of human behaviour due to the coronavirus outbreak. It has a negative impact on economic activity both on the supply and demand side. Firms might have to restrict production and consumers might not be able to go out for shopping, or they may be afraid to travel. To approximate the extent of social distancing, two measures have been proposed.

The Oxford stringency index is rank scaled and built on different components, such as the closing of schools and workplaces, the cancellation of public events, restrictions on gatherings and public transport, stay-at-home regulations and limitations on domestic and international travel activities (Hale et al., 2020). The composite measure is obtained as the arithmetic average of eight individual indicators, rescaled to vary from 0 to 100. Increasing values of the index imply stricter regulations.[2]

The Google mobility reports (Google LLC, 2020) are based on the number of searches across different types of destinations such as retail and recreation, public transport, parks, supermarkets and pharmacies, workplaces and residential areas. Higher values indicate increasing search activities. The series in these reports are created with aggregated, anonymised sets of data from Google users who have turned on the Location History setting, which is off by default. It should be noted that the number of searches is not an indicator of past mobility patterns. Instead, it can be viewed as an indicator for the interest in movement. In this sense, it has leading properties for the impact of the crisis. Not surprisingly, with a coefficient of -0.25, the correlation between the series is not overwhelmingly high.[3]

The two indices reflect public and private reactions to the crisis (Gros et al., 2020). The Oxford indicator is broader as it reflects actual social distancing restrictions of all kinds, not only related to movement. Both indicators are reported at the daily frequency. To investigate their potential impact on the economy, monthly averages are considered (Figure 2, with data for the four largest EU countries). The strength of restrictions declined from its peak in April, but as of August, a high level of interventions is still in place.

1 Due to strong collinearities between the regressors in short time series, fixed time effects are not included. The pandemic led to rapid adoptions of policy interventions across heterogeneous countries that can be explained by a leader-follower approach, at least partially. The stringency indicators reflect the co-movements of government actions, also driven by uncertainty among policymakers (Sebhatu et al., 2020).

2 It may be argued that the Oxford stringency index does not exploit the information from certain policies in an optimal way. Using principal components instead of simple averages shows that some measures should be less weighted than others, depending on the country considered. For instance, international travel restrictions receive a lower weight in France and Italy, but not in Germany and Spain.

3 As an alternative to the Google index, Apple reports on mobility trends: https://covid19.apple.com/mobility. They are less complete than from Google, as they only show the category of transportation, where driving, walking and transit are distinguished.

Figure 3
**Economic sentiment indicator and GDP growth (y-o-y) in Germany and France**

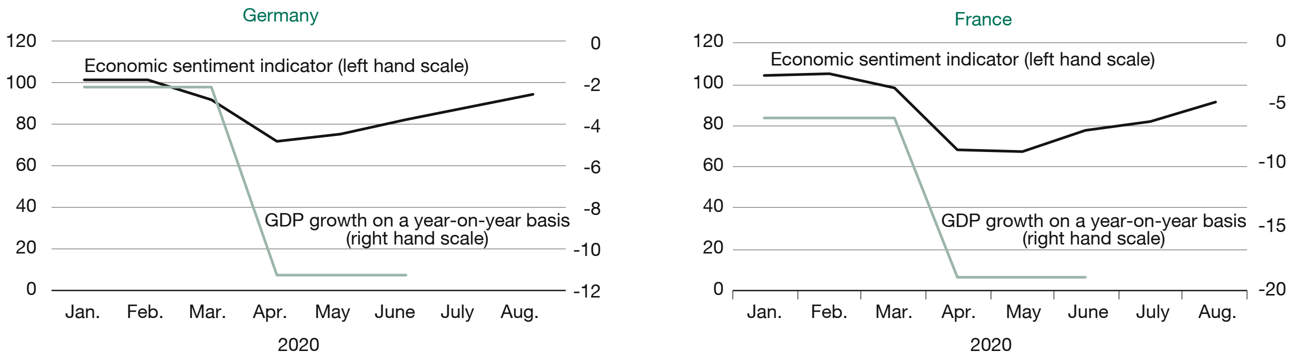

Source: European Commission, 2020.

Except for Spain, the Google index is currently higher than before the crisis, probably driven by catch-up effects in the last few months. This might reflect that some components like retail and recreation and parks exhibit a strong seasonal pattern. While the indices may be low in winter, they are expected to rise in summer, independent of the state of outbreak of the virus. Therefore, the upward trend at the end of the sample could be exaggerated.

## Monthly indicators for economic activity

One critical aspect for policymakers is the ability of the indicators to trace the economic downturn. While data on infections and restrictions are available on a daily basis, actual macroeconomic data (as opposed to the many real time partial indicators, such as freight volumes, cinema attendance, etc.) are available at much lower frequencies. For example, GDP is reported per quarter for EU countries. Thus, it cannot show the fast-moving impact of the pandemic and the recovery (Figure 3).

The information for the second quarter (April to June) hides the nascent recovery, which set in already in May and even more in June. In fact, the growth rate of production, measured on a monthly basis, changed its sign during this quarter. A similar observation applies to the first quarter, where the reduction is mainly due to a very steep fall in March. Thus, the switch to the monthly frequency is highly recommended. Unemployment is reported monthly, but compared to output, the labour market is more persistent in Europe and widespread recourse to short-time working measures throughout the EU limited the impact of the recession on the labour market.

To measure economic activity on a monthly basis, two indicators are selected. Industrial production growth shows actual production but covers only the manufacturing sector (including energy). Services, the largest part of GDP, are not taken into account by this indicator. This is a major drawback, as policies of social distancing are often targeted at services, such as limitations to travel and restaurants. As a rule, industrial production reacts rather fast to a changing macroeconomic environment. The variable is an important tool to predict GDP at many institutions, including central banks.

The economic sentiment index (ESI) taken from the EU Commission (2020) serves as a proxy for the change in contemporaneous GDP, compared to the previous year. Unlike GDP, the ESI is available at the monthly frequency.[4] It is based on regular harmonised surveys for different economic sectors in EU countries. Figure 3 shows, as an example, the additional information that the monthly ESI can deliver compared to quarterly GDP for France and Germany.

Answers to the questionnaire are measured on an ordinal scale. The surveys are carried out at the national level by certain institutions, including ministries, statistical offices, central banks, research institutes or private companies. They are conducted according to a common methodology, which consists essentially of harmonised questionnaires. Sectors included in the ESI are industry (40%), services (30%), consumers (20%), retail (5%) and construction (5%). Based on quarterly data and a longer time

4 The purchasing managers index (PMI) is also available at the monthly frequency. However, it is highly correlated with the ESI and yields little additional information.

horizon (1999.1-2020.2) the correlation between the ESI and contemporaneous GDP growth is about 0.9 on the EU average (European Commission, 2017). It should be noted that the two measures of economic activity are growth rates and stationary. They do exhibit deterministic or stochastic trends during the sample period. Hence, the risk that the results are driven by spurious correlations can be neglected.[5] Since the short-run fluctuations of economic activity are taken as endogenous variables, fundamental models to explain their trends are not required.

As an alternative, the IMF (2020) used daily and weekly indicators of economic activity, namely the Google index and the number of job postings published on the website Indeed. However, it is an open question whether mobility or job advertisements can really be taken as proxies of economic activity.

## Results of the empirical analysis

Panel models with country fixed effects are estimated for 27 EU countries and the period of the pandemic, February to August 2020. Overall, 27 x 7 = 189 potential data points are available, implying a sufficiently high number of degrees of freedom. Due to gaps in the data for several small economies like Cyprus, Malta and Croatia, the actual number of observations is slightly lower. The two indicators of economic activity are explained by the Oxford and Google indicators. The fixed effects control for different national structures, different fiscal policy measures and any other variables that are fairly constant in the short run. In principle, the explanatory power should be higher for the ESI, as services are mostly targeted by the restrictions.

The results are in line with expectations (Table 1). The most important variable seems to be the Oxford stringency index. The point estimate is negative in all specifications and significant at the 0.1% level. Higher restrictions are correlated negative with output growth. This result is not surprising, as the indicator is based on the actual lockdown measures.

The results are mixed for the Google indicator. In the regression for industrial production we find the expected positive sign. However, for ESI we find a negative sign. This would mean that higher movement is correlated with less growth. We relate this – at first sight – counter-intuitive finding to our observation that the Google indicator does not measure movement, but searches. People might search more if they are forced to stay close to home. At any rate, there are indications that this is a spurious result. The coefficient of determination is slightly lower in the extended specification (0.708 instead of 0.733), and standard error is higher. This suggests to us that the Google indicator does not constitute a good proxy for economic activity.

Table 1

**Panel regressions explaining the recession and recovery**

| Dependent: Economic sentiment indicator (ESI) | | | |
|---|---|---|---|
| OXFORD | -0.421 (0.021)*** | | -0.508 (0.034)*** |
| GOOGLE | | -0.227 (0.056)*** | -0.141 (0.043)*** |
| FE | 103.9 (5.0) | 83.7 (3.8) | 107.7 (4.7) |
| R2 | 0.733 | 0.286 | 0.708 |
| SER | 8.053 | 13.127 | 8.233 |
| **Dependent: Growth in industrial production (GIP)** | | | |
| OXFORD | -0.291 (0.023)*** | | -0.245 (0.033)*** |
| GOOGLE | | 0.452 (0.048)*** | 0.243 (0.048)*** |
| FE | 2.3 (4.6) | -8.6 (4.5) | 2.1 (4.2) |
| R2 | 0.657 | 0.622 | 0.751 |
| SER | 8.036 | 8.141 | 7.219 |

Note: Panel regression with 25 (ESI) and 22 countries (GIP), 2020.2-2020.8 (leading to 154 and 175 observations respectively), country fixed effects (FE). Entries in FE indicate average fixed effect, standard deviation of fixed effects in parentheses. R2=Coefficient of determination, SER standard error of regression. Numbers denote regression coefficients, standard errors in parentheses.

Source: Authors' estimation.

In the political debate, it is often argued that a worsening of the coronavirus outbreak (higher new infections or fatalities) might impact the recovery because people would become more cautious, reducing some activities. However, an independent effect of the human losses on economic activities is not confirmed in our analysis. In Europe, the recovery continued despite a rise in infections during the summer.[6] It is possible, however, that the reported fatalities could have an independent impact on the willingness of people to move or to consume. The number of fatalities is significant at the 0.05 level in three out of the four specifications, and it is significant at the 0.1 level in the fourth

5 Deb et al. (2020) employed daily global data on real-time containment measures and indicators of economic activity such as Nitrogen Dioxide ($NO_2$) emissions, flights, energy consumption, maritime trade and mobility indices. Here again, the problem is that the correlation between these real-time indicators and broader economic outcome remains untested.

6 Including the number of infections (per 1000 inhabitants) as an explanatory variable besides the Oxford index yielded a positive sign (more infections, better economic performance) but the effect is usually not significant. Results are available from the authors upon request.

Table 2
### Extensions of panel regressions by human losses

| Dependent: Economic sentiment indicator (ESI) | | | |
|---|---|---|---|
| OXF | -0.514 (0.035)*** | -0.504 (0.034)*** | -0.518 (0.034)*** |
| GOO | -0.138 (0.043)** | -0.162 (0.045)*** | -0.176 (0.044)*** |
| COI | 0.001 (0.001) | | 0.004 (0.002) |
| COD | | -0.015 (0.010) | -0.032 (0.012)** |
| FE | 107.6 (4.6) | 107.8 (4.9) | 107.7 (4.5) |
| R2 | 0.710 | 0.713 | 0.723 |
| SER | 8.243 | 8.200 | 8.082 |
| **Dependent: Growth in industrial production (GIP)** | | | |
| OXF | -0.233 (0.032)*** | -0.237 (0.031)*** | -0.238 (0.032)*** |
| GOO | 0.201 (0.050)*** | 0.169 (0.050)*** | 0.169 (0.050)*** |
| COI | -0.003 (0.001)*** | | 0.001 (0.002) |
| COD | | -0.030 (0.008)*** | -0.034 (0.013)** |
| FE | 2.3 (3.9) | 2.2 (4.0) | 2.2 (4.1) |
| R2 | 0.765 | 0.779 | 0.779 |
| SER | 7.038 | 6.833 | 6.861 |

Note: Panel regression with 25 (ESI) and 22 countries (GIP), 2020.2-2020.8 (leading to 154 and 175 observations respectively), country fixed effects (FE). Entries in FE indicate average fixed effect, standard deviation of fixed effects in parentheses. OXF=Oxford stringency index, GOO=Google mobility indicator, COI=Number of new infections, COD=Deaths related to the coronavirus. R2=Coefficient of determination, SER standard error of regression. Numbers are regression coefficients with standard errors in parentheses, *** and ** denote significance at the 0.01 and 0.05 level of significance.

Source: Authors' estimation.

regression (Table 2). However, its inclusion contributes only little to the overall performance of the equation.

Overall, these results confirm the general rule that the severity of the outbreak itself should not be considered as a good predictor of the output loss given the 'paradox of prevention': A country that implements stringent social distancing measures early and thus prevents any spread of the disease might experience an initial sharp decline in economic activity. However, the hypothesis that news about the number of infections has an impact on consumer confidence and demand in addition to the impact of social distancing restrictions, cannot be broadly confirmed.

A natural extension is to analyse separately the individual elements of the composite Oxford stringency index. All of the individual components display a significant negative correlation to economic activity. The t-values often exceed 10 in absolute value, especially in the ESI regressions (Table 3). Since all these sub-indicators are measured on a scale from 0 to 100, one can use the point estimate of the coefficient to measure the impact of specific types of restrictions on the economy. We find the highest coefficient for 'stay-at-home regulations', i.e. the limitations to leave houses, apart from pre-defined exceptions for work and grocery shopping.[7] Other elements of social distancing policies appear to be slightly less costly in economic terms, suggesting that they should be favoured by policymakers if new restrictions are actually required.

Table 3
### Impact of different non-pharmaceutical measures on economic activity

| Type of measure | ESI | GIP |
|---|---|---|
| School closing | -0.303 (0.031)*** | -0.148 (0.027)*** |
| Workplace closing | -0.316 (0.034)*** | -0.181 (0.027)*** |
| Cancel public events | -0.247 (0.026)*** | -0.134 (0.020)*** |
| Restrictions on gathering | -0.294 (0.024)*** | -0.148 (0.021)*** |
| Close public transport | -0.343 (0.049)*** | -0.161 (0.039)*** |
| Stay-at-home regulations | -0.392 (0.057)*** | -0.236 (0.044)*** |
| Domestic travel | -0.214 (0.033)*** | -0.101 (0.027)*** |
| International travel | -0.332 (0.032)*** | -0.158 (0.025)*** |

Note: See notes to Table 2. Entries denote coefficients of particular measures in regressions using either ESI or GIP as dependent variable. Standard errors in parentheses, country fixed effects. Google mobility indicator and the number of fatalities serve as controls.

Source: Authors' estimation.

## Conclusions

Any analysis of the economic impact of the COVID-19 pandemic can only be undertaken in a 'sea of endogeneity'. The initial imposition of social distancing measures might be considered exogenous. However, their time path might have been influenced by the actual fall in output that followed. Fiscal policy also reacted strongly in most countries to the expected fall in output, possibly mitigating some of the economic losses. Given all these potential confounding factors, our results need to be interpreted with caution. Strictly speaking, the correlations we uncover represent correlations, not causality.

An interesting pattern emerges nevertheless. Social distancing restrictions, as measured by the Oxford stringency indicator, constitute the one variable that is most tightly correlated with the recession and recovery across EU member states. In contrast, the state of the outbreak

7 Deb et al. (2020) arrive at a similar conclusion that workplace closures and stay-at-home orders are associated with the largest economic costs.

of the coronavirus represents only a marginal factor: The number of new infections is not significant, and the number of fatalities is only weakly related to the state of the economy.

All the individual components embedded in the composite Oxford indicator are highly negatively correlated with economic activity. But the introduction of stay-at-home regulations stands out as having the strongest effect. Many medical studies concluded that stay-at-home restrictions are less important for the spread of the virus. This would imply that this type of measure has a low benefit/cost ratio and should be avoided. Governments might do better focusing on other components of social distancing, such as restrictions on public events and public gatherings.

Our results also suggest, subject to the caveats mentioned above, that a rise in infections should not per se endanger the recovery unless it leads governments to tighten social distancing requirements. Increases in the number of fatalities seem to be only weakly correlated with the state of the economy, least compared with the degree of social distance restrictions imposed by governments.
